# Resilience in surgery

**DOI:** 10.1002/bjs.11493

**Published:** 2020-03-04

**Authors:** A. Arnold‐Forster

**Affiliations:** ^1^ Department of Humanities University of Roehampton London SW15 5PU UK

The past 5 years has seen an increased debate about the importance of resilience in surgery. Resilience is defined by *Psychology Today* as ‘…that ineffable quality that allows some people to be knocked down by life and come back stronger than ever; the capacity to recover quickly from difficulties, often equated with toughness’^1^. It is a type of emotional armour, a shield that defends people from excessive and negative feeling.

In 2002, Diane Coutu^2^ wrote an article for the *Harvard Business Review* entitled ‘How Resilience Works’. Fifteen years later, in a blog post for the *Association for Academic Surgery*, Dawn Coleman applied Coutu's account of resilience to surgery. Coleman^3^ quoted from Coutu's article: ‘more than education, more than experience, [and] more than training, a person's level of resilience will determine who succeeds and who fails. That's true in the cancer ward, it's true in the Olympics, and it's true in the boardroom’. Coleman^3^ went on: ‘I pose to you…that it is also true in surgery’.

The idea that surgeons need to be resilient is prevalent. Prominent voices in the profession suggest that it is not only something surgeons should possess, but that it is also something that they can learn and develop. The Royal College of Surgeons of Edinburgh^4^ writes on its website: ‘Resilience is now recognised in healthcare as a collection of features that can be learned by individual doctors’^4^. It offers its members a simple ten‐step programme and has published ‘expert tips for resilience’ on its website. Tips include: try to maintain a positive outlook, and find an exercise regimen you'll stick to (without offering any suggestions about how readers might maintain a positive outlook or fit exercise around night shifts). Hospitals are now papered with posters that implore staff to be optimistic and never give up. It is unclear where the evidence for these interventions comes from, or even what resilience is, beyond the capacity to cope with the stresses and strains of a surgical career.

Before 2000, resilience as a notion was barely mentioned in surgical literature, and used only to refer to patients. People with serious illnesses or injuries were the resilient ones, not the professionals who cared for them. Resilience rhetoric, therefore, emerged in response to the specific social, cultural and political context of the late 1990s. In 1997, the Labour government won a landslide general election. They introduced the European Working Time Directive (EWTD), which entered European law in 1998 and limited the hours of work a surgeon could undertake^5^. A key component of the EWTD was that the maximum period of work for a junior doctor without rest was 13 h.

Many young surgeons argue that the shorter sessions of work mandated by the EWTD led to complex rotas and frequent handovers, with implications for both patient safety and professional well‐being. Before the EWTD, long working hours were made bearable by the emotional support provided by colleagues, and the compassionate connections they could form with their patients when able to maintain continuity of care. The result was that informal support networks within the surgical profession and the hospital have, over the past 30 years, collapsed.

Previously, in the UK and elsewhere, surgeons trained as part of a firm, a hierarchical structure of senior and less‐senior practitioners. They often lived in, or very close to, the hospital and subscribed to a culture of overwork and surgical heroism, with everyone making excessive time commitments to their job. Nostalgia can obscure the reality of past working lives; however, there are features of mid‐20th century surgical life that likely sustained the emotional health of healthcare professionals and supported resilience. Now, increased workload, more frequent handovers, staff shortages and restricted resources intensify the stresses and strains of surgical life; at the same time, there has been a decline in informal support structures that are not being replaced by formal interventions designed to support emotional and mental ill health. Community, professional bonds and social interaction were key features of past surgical life. These are now superseded by a simplistic and individualist notion of resilience that identifies the internal workings of the psyche as both source and solution to distress.

As Boyle and colleagues^6^ show in this issue of BJS, it is often surgeons' emotional investment in their patient's health that has the potential to cause the greatest harm to their well‐being. Deaths after surgery are infrequent, but can have profound emotional consequences and become the source of lasting grief and regret. Although there is little critical appraisal of what resilience is, whether it is something that is desirable for a surgeon to have, or even learn, surgical deaths are an example of when resilience is most needed.

Resilience has a loose and imprecise definition in many current surgical conversations, and its capaciousness can render it almost meaningless. As yet, there is no convincing way to assess an individual's resilience, nor any way to assess the efficacy of interventions designed to improve it. A resilience training programme could frame negative emotions as damaging or obstructive. Perhaps most troublingly, individualistic notions of resilience frequently take the place of vital structural critiques and interventions, as well as evidence‐based solutions such as Schwartz Rounds^7^.

There are occasions where resilience might prove useful to the surgeon. Boyle and colleagues^6^ not only identify surgical death as a cause of emotional distress for surgeons, they also make recommendations for strategies to increase resilience and manage regret. They advocate better communication with patients, families, colleagues and at handover as a source of ‘reflective change to…reduce postdecision regret’. They also identify a need to prioritize non‐technical skills training for surgeons to improve resilience and manage professional well‐being. These suggestions are critical for three reasons. First, because they do not seek to exclude emotions from surgical practice and they do not see feelings as obstructions to technical skill or good surgical care. Second, because they identify the importance of communication and social support rather than individualizing resilience. Third, and most importantly, they focus on long‐term and structural changes to surgical training rather than emphasizing short‐term, small‐scale interventions like exercise classes and attitudinal change.

These are crucial lessons to learn about resilience. Inadequate working conditions, mental illness and emotional distress in surgery will not be solved by posters imploring practitioners to be optimistic! Instead, research is needed that identifies the sources of emotional harm and professional dissatisfaction. Improving surgical resilience will require innovative interventions that remain alert to the supportive nature of workplace communities, emphasize communication and the patient experience, and engage with the political and economic realities of the 21st century world and workforce.
